# Intersectional Stigma and Barriers to Mental Health Among Older Adults With HIV in San Francisco

**DOI:** 10.1093/geroni/igaa057.2562

**Published:** 2020-12-16

**Authors:** Paul Nash, Mark Brennan-Ing, Tonya Taylor, Stephen Karpiak

**Affiliations:** 1 University of Southern California, Los Angeles, California, United States; 2 Hunter College, CUNY, New York, New York, United States; 3 SUNY Downstate Health Sciences University, Brooklyn, New York, United States; 4 ACRIA Center on HIV & Aging at GMHC, new York, New York, United States

## Abstract

For older adults with HIV, forms of privilege and oppression (racism, poverty, limited access to quality education, and inequalities in criminal justice system) intersect with stigmatized social identities (immigrant status, non-cisgender identity, sexual orientation, depression, and addiction) that may increase cumulative burden of psychological distress, contribute to poor clinical outcomes, and create disparities in health care utilization. Using survey and focus group data from the San Francisco ROAH 2.0 (Research on Older Adults with HIV) site, we explored how layered intersectional identities (minority affiliation, gender and sexual orientation), life experiences (immigration, trauma) and forms of systemic oppression (poverty, low educational attainment, and incarceration) impact the utilization of mental health supportive services. Immigrants, minority women, and heterosexual men had higher burdens of depression compared to their white counterparts. Similarly, inhabiting multiple stigmatized identities resulted in both low and variable levels of mental health care utilization, suggesting need for targeted intervention efforts.

